# Grading of cytokine release syndrome associated with the CAR T cell therapy tisagenlecleucel

**DOI:** 10.1186/s13045-018-0571-y

**Published:** 2018-03-02

**Authors:** David Porter, Noelle Frey, Patricia A. Wood, Yanqiu Weng, Stephan A. Grupp

**Affiliations:** 1 Division of Hematology-Oncology, Blood and Marrow Transplantation and Cellular Therapy Program, Perelman School of Medicine and Abramson Cancer Center, University of Pennsylvania, Philadelphia, PA USA; 2 Novartis Pharmaceuticals Corporation, East Hanover, NJ USA; 3 Department of Pediatrics, Children’s Hospital of Philadelphia, University of Pennsylvania, Philadelphia, PA USA; 4 Division of Oncology, Center for Childhood Cancer Research and Cancer Immunotherapy Program, Children’s Hospital of Philadelphia, 3501 Civic Center Blvd. CTRB 3006, Philadelphia, PA 19104 USA

**Keywords:** CAR T cell therapy, Cytokine release syndrome, Safety

## Abstract

**Background:**

Anti-CD19 CAR T cell therapy has demonstrated high response rates in patients with relapsed or refractory (r/r) B cell malignancies but is associated with significant toxicity. Cytokine release syndrome (CRS) is the most significant complication associated with CAR T cell therapy, and it is critical to have a reproducible and easy method to grade CRS after CAR T cell infusions.

**Discussion:**

The Common Terminology Criteria for Adverse Events scale is inadequate for grading CRS associated with cellular therapy. Clinical experience with the anti-CD19 CAR T cell therapy tisagenlecleucel at the University of Pennsylvania (Penn) was used to develop the Penn grading scale for CRS. The Penn grading scale depends on easily accessible clinical features; does not rely on location of care or quantitation of supportive care; assigns grades to guide CRS management; distinguishes between mild, moderate, severe, and life-threatening CRS; and applies to both early-onset and delayed-onset CRS associated with T cell therapies. Clinical data from 55 pediatric patients with r/r B cell acute lymphoblastic leukemia and 42 patients with r/r chronic lymphocytic lymphoma treated with tisagenlecleucel were used to demonstrate the current application of the Penn grading scale.

**Conclusion:**

We show that the Penn grading scale provides reproducible CRS grading that can be useful to guide therapy and that can be applied across clinical trials and treatment platforms.

## Background

### CAR T cell therapy

Chimeric antigen receptor (CAR) T cell therapy is a novel immunotherapeutic approach for treating cancer, with exciting initial successes targeting CD19 malignancies. CARs are synthetic molecules that combine into a single functional receptor containing the following components: an antigen recognition domain, one or more T cell costimulation domain(s), and, typically, the CD3ζ T cell activation domain [1, 2]. Gene transfer technology is used to genetically modify autologous or allogeneic T cells to contain the CAR of interest. Expression of the CAR reprograms the T cell, combining tumor specificity with a potent cytotoxic immune response [3–5]. CARs targeted at CD19, a cell surface protein broadly expressed across the normal B cell lineage and on most B cell malignancies, can induce profound and durable tumor responses in populations of patients with relapsed or refractory (r/r) disease. For example, in patients with r/r acute lymphoblastic leukemia (ALL), CD19 CAR T cell therapies have shown complete response (CR) rates of ≈ 90% in single-center trials and ≈ 70–80% in multicenter trials [6–11]. In patients with r/r non-Hodgkin lymphoma (NHL), CR rates of ≈ 50–70% have been reported with CD19 CAR T cell therapies [12–15]. CR rates in patients with r/r chronic lymphocytic leukemia (CLL) receiving CD19 CAR T cells were ≈ 30–50% [16, 17], with some remissions lasting beyond 6 years.

The most prominent and serious toxicity of CAR T cell therapy is cytokine release syndrome (CRS), an acute inflammatory process marked by a spectrum of clinical symptoms and substantial but transient elevations of serum cytokines [18, 19]. Centers administering CAR T cells have developed and employed different CRS grading scales, making it difficult to compare CRS severity and outcomes, with and without interventions, between studies. CRS associated with other therapeutics has been graded by the National Cancer Institute Common Terminology Criteria for Adverse Events (CTCAE) system; however, this system is inadequate for cellular therapeutic approaches. The definitions of CRS, CRS onset, and CRS resolution are also not clearly or uniformly defined. A more broadly applicable CRS grading scale with onset and resolution criteria and with improved granularity would greatly benefit the oncology community.

An alternate CRS grading scale from the University of Pennsylvania (Penn grading scale) is described in this review. Clinical safety data from patients receiving tisagenlecleucel (formerly CTL019), an anti-CD19 CAR T cell therapy developed at Penn and the Children’s Hospital of Philadelphia (CHOP), is presented to demonstrate the utility of this CRS grading scale in different disease settings and age groups. This Penn grading scale has been applied to CLL, ALL, and diffuse large B cell lymphoma (DLBCL) [7, 16]; has been implemented in numerous multicenter, global clinical trials across North America, Europe, Asia, and Australia [8, 9, 20, 21]; and is being used to grade CRS associated with several different types of CAR T cell therapies, including CAR T cells targeting antigens other than CD19 and humanized CAR T cell therapies [22, 23]. This CRS grading scale is based on data collected from this cohort of 125 adult and pediatric patients treated with tisagenlecleucel.

### CRS: a clinical overview

The term CRS has been occasionally used interchangeably with the term “cytokine storm.” Although these conditions share a similar clinical phenotype and biomarker signature, they have distinct characteristics (Table 1). In general, the term cytokine storm was coined to reference situations in which the immune system is activated independent of tumor targeting, resulting in overwhelming systemic inflammation and hemodynamic instability, leading to multi-organ dysfunction and fatal outcomes in some cases. In the context of immunotherapies, cytokine storm was described more than 25 years ago in patients receiving OKT3, a monoclonal antibody (mAb) targeting the T cell surface receptor CD3. Cytokine storm was later observed in healthy volunteers receiving the anti-CD28 mAb TGN1412 [24, 25]. With both T cell-activating antibodies, rapid onset of clinical symptoms within minutes or hours was observed, along with activation of the subject’s T cells. Rapid elevation in specific proinflammatory cytokines was described after these therapies; for example, in the early OKT3 studies, patients displayed increased tumor necrosis factor (TNF)-α levels just 1 h after the first infusion [24].

**Table 1:** Comparison of cytokine storm and CRS

	Cytokine storm	CRS
Pathogenesis	Immune system is activated independent of tumor targeting, as with antibodies specific for CD3 or CD28	T cells become activated as they recognize tumor antigen
Timing	T cell activation and clinical symptoms occur within minutes to hours of treatment	Symptoms may be delayed until days or weeks after treatment, depending on the kinetics of T cell activation
Mediators	TNFα and IFNγ are key mediators	IL-6 is a key mediator
Treatment	Symptoms can be resolved using corticosteroids or by stopping the T cell-directed infusion	Symptoms can be resolved using IL-6 pathway inhibition or corticosteroids

*CRS* cytokine release syndrome, *IFN* interferon, *IL* interleukin, *TNF* tumor necrosis factor

In contrast to cytokine storm, the term CRS has been used to describe the specific spectrum of reactions seen after administration of targeted therapies that cause significant activation of the immune system, such as CAR T cells and bispecific T cell-engaging antibodies. CRS is characterized by delayed onset of clinical symptoms, suggesting that the symptoms are a result of on-target, antigen-driven T cell activation, and proliferation. The kinetics of delayed CRS, characteristic of T cell and tumor cell interaction, have also been observed in patients receiving blinatumomab, a bispecific T cell-engaging antibody targeted to CD3/CD19. Although blinatumomab is not an adoptive cellular therapy, its mechanism of action depends on antigen-specific T cell cytotoxicity, accounting for similarities between CRS observed with blinatumomab and CAR T cell therapies. The incidence of blinatumomab-associated CRS is quite variable (2–36%) and may reflect differing prophylactic approaches, underreporting of CRS, and/or variable use of CRS definitions and criteria [26–28]. However, it is important to note that the mode of administration of blinatumomab is designed specifically to minimize CRS. In a recent 189-patient study of blinatumomab, measures to prevent CRS included using prephase dexamethasone in patients with high tumor burden and within 1 h before treatment initiation in each cycle [28, 29]. The blinatumomab starting dose was lower in the first week of treatment than in the subsequent 3 weeks [28]. Furthermore, CTCAE grading is much more useful for an ongoing therapy such as blinatumomab, which can be stopped to mitigate toxicity. In addition, blinatumomab appears to be a much less efficient and less powerful way to activate T cells compared with CAR T cell therapies. The ability to discontinue the drug before prolonged CRS occurs, the use of CTCAE grading, and the potency of the therapy likely contribute to the low rates of CRS observed (2% of patients experienced grade 3 CRS).

Although CRS observed in patients with ALL treated with CD19-targeted CAR T cell therapies varies in severity and kinetics depending on tumor burden [7], there is a clear delay in both CRS-associated clinical symptoms and the related elevation of cytokine levels. In most patients, CRS occurs 1–14 days after CAR T cell infusion and rarely develops more than 17 days after infusion [7, 16, 30]. The data suggest that CAR T cell-associated CRS depends on T cell engagement with the target antigen followed by proliferation and functional response. Other factors influencing CRS may include disease type, nature and degree of lymphodepletion, and possibly CAR design. Because CRS is related to T cell engagement with a target antigen, it is not expected to be restricted to anti-CD19 therapies only. CRS was observed in four of seven (57%) evaluable pediatric or young adult patients with r/r ALL who received CD22-targeted CAR T cells [31]. In addition, CRS has been observed in patients with multiple myeloma (MM) who received CAR T cells targeting CD19 or B cell maturation antigen (BCMA) [23, 32, 33].

Several analyses have shown that IL-6 is likely a central mediator of CRS after CAR T cell therapy, although elevations in other cytokines, such as IFNγ, GM-CSF, IL-5, and IL-8, are also consistently noted. Following our report of the successful use of IL-6 receptor blockade in a patient with ALL and life-threatening CRS [18], the approach of IL-6 signaling blockade has been used broadly to treat patients with CRS [7, 18, 19, 30]. The IL-6 receptor antagonist antibody tocilizumab has been used in numerous cases of severe CRS and results in rapid improvement in clinical symptoms in most patients, without depletion of CAR T cells or apparent attenuation of the therapeutic effect, but further investigation into the durability of remissions is needed. Preclinical modeling suggests that disruption of IL-6 signaling may not impact CAR T cell activity [34], and clinical trials are underway to evaluate outcomes of CAR T cell therapy testing a preemptive strategy of early anti-IL-6-directed intervention for CRS (NCT02906371).

### Current CRS grading systems

Initially, CRS had been graded by the CTCAE grading scale for adverse event reporting (Table 2) [35]; however, this scale was developed before CAR T cell therapy-associated CRS was well understood. With T cell-activating modalities such as CAR T cell therapy, the CTCAE system fails to adequately document the timing, spectrum, and severity of CRS events. For example, CTCAE CRS grading reflects an expected onset of CRS symptoms immediately upon drug infusion—potentially within minutes to hours, which is more consistent with the acute cytokine storm seen with some T cell antibody therapies and fails to reflect the potential for delayed CRS onset after CAR T cell infusion. In addition, the CTCAE system assumes that CRS can be alleviated by stopping an ongoing infusion. Because CAR T cells can expand significantly in vivo and are often administered as a single infusion, stopping the infusion is not a relevant strategy; therefore, determining CRS grade based on response to treatment interruption has no relevance to describing and treating cellular therapy-associated CRS.

**Table 2:** CRS grading scales: Penn grading scale, CTCAE v4.0, and 2014 Lee et al. scale

	Penn grading scale [16]	CTCAE v4.0 [35]	2014 Lee et al. [36]
Grade 1	Mild reaction: treated with supportive care such as antipyretics, antiemetics	Mild reaction; infusion interruption not indicated; intervention not indicated	Symptoms are not life-threatening and require symptomatic treatment only, e.g., fever, nausea, fatigue, headache, myalgias, malaise
Grade 2	Moderate reaction: some signs of organ dysfunction (e.g., grade 2 creatinine or grade 3 LFTs) related to CRS and not attributable to any other condition. Hospitalization for management of CRS-related symptoms, including fevers with associated neutropenia, need for IV therapies (not including fluid resuscitation for hypotension)	Therapy or infusion interruption indicated but responds promptly to symptomatic treatment (e.g., antihistamines, NSAIDs, narcotics, IV fluids); prophylactic medications indicated for ≤ 24 h	Symptoms require and respond to moderate intervention. Oxygen requirement < 40% or hypotension responsive to fluids or low-dose pressors or grade 2 organ toxicity
Grade 3	More severe reaction: hospitalization required for management of symptoms related to organ dysfunction, including grade 4 LFTs or grade 3 creatinine related to CRS and not attributable to any other conditions; this excludes management of fever or myalgias; includes hypotension treated with intravenous fluids (defined as multiple fluid boluses for blood pressure support) or low-dose vasopressors, coagulopathy requiring fresh frozen plasma or cryoprecipitate or fibrinogen concentrate, and hypoxia requiring supplemental oxygen (nasal cannula oxygen, high-flow oxygen, CPAP, or BiPAP). Patients admitted for management of suspected infection due to fevers and/or neutropenia may have grade 2 CRS	Prolonged reaction (e.g., not rapidly responsive to symptomatic medication and/or brief interruption of infusion); recurrence of symptoms following initial improvement; hospitalization indicated for clinical sequelae (e.g., renal impairment, pulmonary infiltrates)	Symptoms require and respond to aggressive intervention. Oxygen requirement ≥ 40% or hypotension requiring high-dose or multiple pressors or grade 3 organ toxicity or grade 4 transaminitis
Grade 4	Life-threatening complications such as hypotension requiring high-dose vasopressors, ^a^ hypoxia requiring mechanical ventilation	Life-threatening consequences; pressor or ventilator support indicated	Life-threatening symptoms. Requirements for ventilator support or grade 4 oxygen toxicity (excluding transaminitis)

*BiPAP* bilevel positive airway pressure, *CPAP* continuous positive airway pressure therapy, *CRS* cytokine release syndrome, *CTCAE* Common Terminology Criteria for Adverse Events, *IV* intravenous, *LFT* liver function test, *NSAID* nonsteroidal anti-inflammatory drug

^a^See specific definition of high-dose vasopressors

#### CTCAE scale

The CTCAE CRS grading scale was then further modified to define mild, moderate, severe, and life-threatening CRS, regardless of the inciting agent, and to guide treatment recommendations [36]. This modified scale uses patients’ response to fluids, vasopressor, and oxygen, as well as their organ toxicities, and was developed for an anti-CD19 CAR T cell program. However, the heavy reliance of this scale on quantification of oxygen support and fluid volumes, which are difficult to standardize and require detailed review to document, introduces additional variability in grading CRS severity across multiple treatment sites [36]. For instance, we find a number of occasions when overnight caregivers order high-flow oxygen or intravenous fluids that are likely in excess of what a patient may actually require, or the response to oxygen support is not well documented. We have thus found it difficult to use quantitative measurements for these kinds of supportive care measures when assessing the severity of illness. This may also pose a challenge in grading CRS in the commercial setting, which may not have research personnel to perform such complex grading.

#### MD Anderson Cancer Center/Lee scale

Recently a group from MD Anderson Cancer Center has, along with other colleagues, proposed a grading system for CRS that is slightly modified from the scale proposed by Lee et al. [37]. These authors have laid out detailed management strategies for CRS and neurologic toxicity based on their grading scale. However, we feel that many of their management recommendations represent institutional preferences, and/or may be specific to the CAR T cell products tested at their centers, and are not necessarily generalizable for all CAR T cell therapy. We have provided some perspective on these issues [38]. We caution against adopting these recommendations for the entire field and feel that our management guidelines provide rigorous, data-driven recommendations while leaving room for clinical judgment where appropriate.

#### CRP-based scale

Another CRS scale uses a combination of clinical features and serum cytokine and C-reactive protein (CRP) levels as criteria for severe CRS [10]. Other scales for reporting CRS severity have been used, including CTCAE grading or binary grading systems based on whether there is a requirement for anti-cytokine therapy or intensive care unit (ICU) admission; however, decisions to administer anti-cytokine therapy or provide ICU level of care are very dependent on the patient, disease, physician, and center and are hard to quantify [6, 39]. Also, what constitutes CRS onset and resolution is not clearly delineated. These CRS grading scales have yet to be fully evaluated across multiple clinical sites in different countries; therefore, the need to further refine CRS grading scales to make them user-friendly persists [40].

### A new CRS grading scale developed based on treating patients with tisagenlecleucel

A new CRS grading scale (Penn grading scale), developed from treatment experience and data on the first 125 patients to receive tisagenlecleucel, was established to better identify tisagenlecleucel-associated CRS severity and accurately guide CRS management across multiple indications [16]. This system was developed to aid consistent reporting in the first-in-human trials of tisagenlecleucel at Penn and CHOP. It subsequently allowed comparison across trials and indications, including multisite and global tisagenlecleucel clinical trials.

A CRS diagnosis is based on several clinical symptoms. CRS typically begins with low-grade fever and/or myalgias but can escalate over several days to include high fever (which may exceed 40.5 °C). Many cases of mild CRS are resolve with minimal intervention. However, CRS can progress to severity, with eventual transient organ dysfunction requiring aggressive intervention and support. CRS can affect many different organ systems, and clinical manifestations have recently been reviewed [41]. Moderate to severe CRS typically involves progressively high fevers, tachycardia, hypotension, and capillary leak, often leading to hypoxia with pulmonary edema and hepatic and/or renal dysfunction; hypofibrinogenemia, with or without bleeding, may also occur. Evaluation and treatment of other concurrent clinical entities (neutropenic sepsis, tumor lysis syndrome, infections, adrenal insufficiency) are critical for managing these patients. Furthermore, using well-defined CRS management guidelines is essential when treating this complex and occasionally life-threatening condition. Such guidelines have been proposed and outlined, although not strictly dependent on CRS grade [41]. Neurological symptoms may also occur during or following CRS, are variable between patients, and may include delirium, agitation, lethargy, aphasia, and seizures. Guidelines for the treatment of neurological symptoms have also been proposed [41]. In our experience, neurological symptoms do not necessarily follow the same time course as systemic CRS symptoms and may not be as responsive to systemic CRS interventions, such as IL-6-directed therapy. Given the unclear pathophysiology contributing to neurological toxicity, neurological events are not incorporated into the Penn grading scale.

In the Penn grading scale (Table 2), CRS onset and resolution are clearly defined. The CRS-onset date is defined by retrospectively assessing the onset of fevers and/or myalgias that are consistent with CRS and not explained by other events (e.g., sepsis). The retrospective definition of CRS onset is critical for data collection and understanding CRS pathophysiology. CRS resolution is the date when the patient is afebrile and off vasopressors, both for 24 h. Medical sequelae may persist beyond CRS and are not considered part of the CRS definition. Grade 1 CRS is defined as a mild reaction that requires supportive care, including antipyretics and antiemetics. In patients with grade 2 CRS, there may be some signs of organ dysfunction, such as grade 2 creatinine or grade 3 liver function test (LFT) results, hospitalization for managing CRS symptoms (which may include management of fevers in setting of neutropenia), or intravenous therapies (such as antibiotics or other medications). Grade 3 CRS is a more severe reaction that requires hospitalization for management of significant organ dysfunction, including grade 4 LFTs or grade 3 creatinine related to CRS and not caused by other conditions. Hospitalization for management of fever, myalgia, or neutropenia does not constitute a diagnosis of grade 3 CRS because patients with grade 2 CRS are generally admitted to the hospital. Grade 3 CRS may also be further defined by the need for supplemental oxygen such as nasal cannula, continuous positive airway pressure, or bilevel positive airway pressure for hypoxia, and/or treatment with intravenous fluids (multiple fluid boluses or continuous hydration) or low-dose vasopressors for hypotension that in the opinion of the clinician is possibly CRS related. Patients with grade 3 CRS may require fresh frozen plasma or cryoprecipitate for coagulopathy. Grade 4 reactions are life-threatening and involve complications such as hypotension requiring high-dose vasopressors as defined by a fixed formula or hypoxia requiring mechanical ventilation (Table 3). The index lesion of severe CRS is unstable hypotension, hence the centrality of fluid and pressor use to the grading scale.

**Table 3:** Definition of high-dose vasopressors

Vasopressor	Dose for ≥ 3 h
Norepinephrine monotherapy	≥ 0.20 mcg/kg/min
Dopamine monotherapy	≥ 10 mcg/kg/min
Phenylephrine monotherapy	≥ 200 mcg/min
Epinephrine monotherapy	≥ 0.10 mcg/min
If on vasopressin	High-dose if vasopressin + norepinephrine equivalent of ≥ 10 mcg/min (using VASST formula)*
If on combination vasopressors (not vasopressin)	Norepinephrine equivalent of ≥ 20 mcg/min (using VASST formula)*

Adapted from Russel et al. with adjustments to accommodate weight-based dosing [42]

*VASST (Vasopressin and Septic Shock Trial) vasopressor equivalent equation: $$ {\displaystyle \begin{array}{l}\mathrm{Norepinephrine}\ \mathrm{equivalent}\ \mathrm{dose}=\left[\mathrm{norepinephrine}\ \left(\mathrm{mcg}/\min \right)\right]+\left[\mathrm{dopamine}\ \left(\mathrm{mcg}/\mathrm{kg}/\min \right)\div 2\right]\ \\ {}+\left[\mathrm{epinephrine}\ \left(\mathrm{mcg}/\min \right)\right]+\left[\mathrm{phenylephrine}\ \left(\mathrm{mcg}/\min \right)\div 10\right]\end{array}} $$

In our experience, the Penn grading scale is practical for evaluating patients for CRS after CAR T cell therapy for several reasons. The Penn grading scale (1) depends on easily accessible clinical features; (2) does not rely on location of care (e.g., ICU), which depends greatly on the treating physician, center, and patient; (3) does not rely on quantitation of supportive care (e.g., fluid volumes for resuscitation or oxygen concentration received) because this is highly variable, time-limited, and not well-controlled or well-documented; (4) assigns grades to help in management decision-making; (5) distinguishes between mild, moderate, severe, and life-threatening CRS; and (6) applies to both immediate-onset CRS (cytokine storm) observed with some anti-T cell antibodies and delayed-onset CRS observed with other T cell-directed therapies, such as bispecific T cell engaging antibodies and adoptive cellular therapies (CAR T cells).

Absolute cutoff values for vital signs are not used in the Penn grading scale because the significance of such values depends on the individual patient. Serum cytokine levels, while supportive of a diagnosis of CRS, are also not used for grading due to lack of timely availability of testing at global clinical sites, variability between platforms and reagents, and lack of quantitative predictive value. Furthermore, vasopressor use in the Penn grading scale is more granularly defined as low dose vs high dose to better distinguish between greater risks of life-threatening events in the latter group, and it is based upon published criteria from ICU-based studies. Because definitions of levels of vasopressor use differ among institutions, a standardized definition of high-dose vasopressors is used based on guidance from intensive care literature [42]. This approach can be reproducibly applied and is independent of differences in patient care among institutions, providers, disease settings, the target antigen of the T cell-directed therapy, or the symptom onset timing. Also, CRS grade is not defined by either the use or timing of anti-cytokine therapy because clinical judgment is required to apply therapeutic intervention based on individual patient reserve and rapidity of CRS clinical course.

In comparing the CRS grading systems (Table 2), several key differences exist between the CTCAE scale and Penn grading scale: (1) although both systems indicate that grade 1 CRS should be treated with supportive care, the CTCAE grading system specifies no other intervention, whereas the Penn grading scale indicates that antipyretics and antiemetics should be used; (2) the Penn grading scale may be more appropriate for patients treated with CAR T cell therapies, which cannot have a dose “interrupted” once the cells have been administered; (3) the Penn grading scale specifies signs of organ dysfunction and hospitalization for CRS-related systems (fevers with associated neutropenia indicate grade 2); and (4) grades 3 and 4 of the Penn grading scale clearly distinguish between truly life-threatening events (e.g., hypoxia requiring mechanical ventilation) and non-life-threatening scenarios that require significant care (e.g., hypoxia requiring supplemental oxygen); (5) the Penn grading scale gives clear guidance on interventional therapies that have been proven effective. Because the index lesion of severe CRS is unstable hypotension, the use of low-dose vs high-dose vasopressors can define clinical groups with different levels of CRS severity [42]. The Penn grading scale uses the more granular definition of vasopressor use to avoid variability resulting from the early use of lower doses of vasopressors by some institutions and practitioners.

Differences in available CRS grading scales result in a wide potential variation in how a single patient would be graded across the grading systems. For example, if a patient with r/r ALL develops hypotension requiring low-dose vasopressors after anti-CD19 CAR T cell therapy, they would be considered grade 3 on the Penn grading scale, grade 4 on the CTCAE scale (due to lack of differentiation between low-dose and high-dose vasopressors in the CTCAE system), and grade 2 on the 2014 Lee et al. scale recently (Table 2). Because CRS grades may guide management strategies, the patient may have a different outcome if graded using different scales.

### Published CRS data using the Penn grading scale

Initial data on CRS using the Penn grading scale in patients receiving tisagenlecleucel at Penn and CHOP were presented previously. In an analysis of 51 patients with ALL (12 adults and 39 pediatric patients), 18 (35%) experienced grade 1/2, 16 (31%) had grade 3, 12 (24%) had grade 4, and 2 (4%) developed grade 5 CRS [43]. Of these 39 pediatric patients treated with tisagenlecleucel, 14 (36%) developed multiple organ dysfunction syndrome, with hepatic and renal dysfunction being the most commonly observed organ dysfunctions [19]. Two subsequent phase 2, multicenter trials of tisagenlecleucel in pediatric and young adult patients with r/r ALL (ELIANA [NCT02435849], ENSIGN [NCT02228096]) also used the Penn grading scale. Patients in the ELIANA trial were treated at 25 sites in 11 countries across North America, Europe, Asia, and Australia; patients in the ENSIGN trial were treated at nine sites in the USA. Despite many of the trial sites having limited or no experience with cellular therapy, the Penn grading scale was easily adopted in both trials. Overall, 79 of 97 patients (81%) who received tisagenlecleucel experienced any-grade CRS, and grade 3 or 4 CRS occurred in 44 of 97 patients (45%) [44].

The Penn grading scale has been applied across several hematologic malignancies and solid tumors. Among the 14 patients with r/r CLL treated in the pilot trial of tisagenlecleucel, grades 1, 2, 3, and 4 CRS were observed in one (7%), two (14%), two (14%), and four patients (29%), respectively, according to the Penn grading scale [16]. In the Penn experience with tisagenlecleucel in r/r NHL, two patients (7%) had grade 3 CRS and two (7%) had grade 4 CRS (*n* = 30) [45]. A multicenter, global phase 2 trial of tisagenlecleucel in adult patients with r/r DLBCL (JULIET; NCT02445248) also used the Penn grading scale at 27 sites in ten countries across North America, Europe, Australia, and Asia. Following an interim data analysis, it was reported that of the 85 patients who received tisagenlecleucel, 48 (57%) experienced CRS, with 14 (17%) and 8 (9%) having experienced grade 3 and grade 4 CRS, respectively [20].

In addition, the Penn grading scale is being applied to trials of non-tisagenlecleucel CARs, including a BCMA-targeted CAR for myeloma [23] in patients with epithelial ovarian cancer treated with anti-mesothelin CAR T cells [46]. In patients infused with CTL119 (humanized anti-CD19 CAR) after prior therapy with murine-derived CD19-targeted CAR T cells, grades 1–3 CRS, per the Penn grading scale, occurred in four of eight patients (50%) and correlated with efficacy and disease burden similar to our experience with tisagenlecleucel; no grade 4 CRS was observed after retreatment with CTL119 [22]. This experience shows that it is feasible and appropriate to expand the use of the Penn grading scale beyond tisagenlecleucel and may establish this scale as a standard for CAR T cell therapies as well as other T cell-directed therapies developed in the future, including CAR T cell therapies that are humanized or have target antigens other than CD19.

### Clinical application of the Penn grading scale and tisagenlecleucel: CRS data and preliminary observations

Clinical data from 55 pediatric patients with r/r ALL (NCT01626495) and 42 patients with r/r CLL (NCT01747486, NCT01029366) were reviewed for maximum CRS grade, CRS-related events, and interventions. The data are tabulated in Tables 4 and 5, respectively, as a demonstration of the current application of the Penn grading scale. The distribution of maximum CRS grade-defining events matches the criteria for maximum CRS grading by the Penn grading scale in both pediatric ALL and CLL datasets. The Penn grading scale demonstrates how patients with grade 2 vs grade 3 vs grade 4 CRS differ, as supported by the differences in time to onset and duration of CRS, duration of high fevers, frequency of hypotension requiring vasopressors, and need for anti-cytokine therapy. Use of this CRS grading scale distinguishes patient groups that are clearly clinically different in the duration of CRS, duration of fevers, duration of ICU visit, frequency of hypotension requiring vasopressors, requirement for oxygen supplementation, need for anti-cytokine therapy, and need for multiple doses of anti-cytokine therapy. Therefore, the Penn grading scale, using standard clinical variables, delineates and differentiates between patients who differ in the level of medical intervention they need, their duration of CRS, and their risk of organ failure requiring intervention.

**Table 4:** CRS by Penn grading scale grade after tisagenlecleucel infusion in non-CNS3 pediatric patients with ALL

	No CRS	Grade 1	Grade 2	Grade 3	Grade 4
Number of patients, *n* (%)	6 (10.9)	3 (5.5)	23 (41.8)	10 (18.2)	13 (23.6)
Among patients with CRS*					
CRS grade-defining events					
Hypotension that required intervention, *n* (%)	–	0	1 (4.3)	7 (70.0)	12 (92.3)
High-dose vasopressors used, *n* (%)	–		0	0	9 (69.2)
Oxygen supplementation given, *n* (%)	–	0	0	3 (30.0)	12 (92.3)
Patient intubated, *n* (%)	–	0	0	0	6 (46.2)
Duration (days)					
Mean (SD)	–	–	–	–	16.2 (23.24)
Median (range)	–	–	–	–	7.5 (3.0–63.0)
Disseminated intravascular coagulation observed, *n* (%)	–	0	0	0	7 (53.8)
Bleeding observed, *n* (%)	–	–	–	–	4 (30.8)
Blood product support given for bleeding, *n* (%)	–	–	–	–	5 (38.5)
CRS timing					
Time to onset of CRS (days)					
Mean (SD)	–	6.0 (4.36)	5.2 (2.90)	3.7 (2.41)	2.0 (0.58)
Median (range)	–	4.0 (3.0–11.0)	5.0 (1.0–11.0)	2.5 (1.0–7.0)	2.0 (1.0–3.0)
Duration of CRS (days)					
Mean (SD)	–	6.0 (2.00)	4.7 (2.43)	8.2 (3.74)	11.2 (2.03)
Median (range)	–	6.0 (4.0–8.0)	4.0 (2.0–10.0)	7.0 (5.0–18.0)	11.0 (7.0–15.0)
Other CRS-associated events					
High (> 38.3 °C) fevers, *n* (%)	–	1 (33.3)	20 (87.0)	10 (100)	13 (100)
Duration (days)					
Mean (SD)	–	4.0	4.7 (2.60)	7.4 (3.72)	8.1 (2.72)
Median (range)	–	4.0 (4.0–4.0)	5.0 (1.0–10.0)	7.0 (3.0–17.0)	8.0 (4.0–13.0)
Admitted to ICU, *n* (%)	–	0	0	7 (70.0)	13 (100)
Time to ICU admission (days)					
Mean (SD)	–	–	–	5.7 (2.29)	5.8 (1.86)
Median (range)	–	–	–	7.0 (2.0–8.0)	6.0 (3.0–10.0)
Duration of ICU stay (days)					
Mean (SD)	–	–	–	4.0 (2.65)	16.2 (16.42)
Median (range)	–	–	–	3.0 (1.0–9.0)	11.0 (4.0–68.0)
Patient dialyzed, *n* (%)	–	0	0	0	0
Pulmonary abnormalities, *n* (%)	–	0	0	0	6 (46.2)
Anti-cytokine therapy					
Systemic anti-cytokine therapy given, *n* (%)	–	0	0	3 (30.0)	13 (100)
Tocilizumab	–	–	–	2 (20.0)	13 (100)
1 dose	–	–	–	2 (20.0)	8 (61.5)
2 doses	–	–	–	0	5 (38.5)
Corticosteroids	–	–	–	2 (20.0)	7 (53.8)
Other	–	–	–	1 (10.0)	1 (7.7)

Only the first CRS episode is summarized for each patient. Time to onset of CRS is since the first tisagenlecleucel infusion. Time to ICU admission is since first tisagenlecleucel infusion

Patients: *n* = 55; study: NCT01626495, B2101J

*ALL* acute lymphoblastic leukemia, *CRS* cytokine release syndrome, *ICU* intensive care unit, *SD* standard deviation

*All percentages are based on the number of patients with corresponding CRS grades

**Table 5:** CRS by Penn grading scale grade after tisagenlecleucel infusion in adult patients with CLL

	No CRS	Grade 1	Grade 2	Grade 3	Grade 4
Number of patients, *n* (%)	24 (57.1)	2 (4.8)	7 (16.7)	4 (9.5)	5 (11.9)
Among patients with CRS*					
CRS grade-defining events					
Hypotension that required intervention, *n* (%)	–	0	0	2 (50.0)	5 (100)
High-dose vasopressors used, *n* (%)	–	–	–	0	3 (60.0)
Oxygen supplementation given, *n* (%)	–	0	0	2 (50.0)	5 (100)
Patient intubated, *n* (%)	–	0	0	0	2 (40.0)
Duration (days)					
Mean (SD)	–	–	–	–	21.0 (16.97)
Median (range)	–	–	–	–	21.0 (9.0–33.0)
Disseminated intravascular coagulation observed, *n* (%)	–	0	0	0	0
Bleeding observed, *n* (%)	–	–	–	–	–
Blood product support given for bleeding, *n* (%)	–	–	–	–	–
CRS timing					
Time to onset of CRS (days)					
Mean (SD)	–	8.0 (8.49)	7.3 (4.39)	22.5 (32.59)	2.4 (3.13)
Median (range)	–	8.0 (2.0–14.0)	9.0 (1.0–13.0)	9.0 (1.0–71.0)	1.0 (1.0–8.0)
Duration of CRS (days)					
Mean (SD)	–	5.0 (5.66)	9.7 (4.68)	11.3 (4.11)	14.0 (5.43)
Median (range)	–	5.0 (1.0–9.0)	9.0 (3.0–17.0)	12.0 (6.0–15.0)	12.0 (10.0–23.0)
Other CRS-associated events					
High (> 38.3 °C) fevers, *n* (%)	–	0	5 (71.4)	4 (100)	5 (100)
Duration (days)					
Mean (SD)	–	–	8.4 (2.51)	5.5 (3.32)	9.2 (3.27)
Median (range)	–	–	9.0 (6.0–12.0)	5.0 (2.0–10.0)	10.0 (4.0–13.0)
Admitted to ICU, *n* (%)	–	0	1 (14.3)	3 (75.0)	5 (100)
Time to ICU admission (days)					
Mean (SD)	–	–	19.0	28.3 (38.08)	5.6 (4.04)
Median (range)	–	–	19.0 (19.0–19.0)	11.0 (2.0–72.0)	3.0 (2.0–10.0)
Duration of ICU stay (days)					
Mean (SD)	–	–	4.0	5.3 (1.15)	12.8 (11.26)
Median (range)	–	–	4.0 (4.0–4.0)	6.0 (4.0–6.0)	9.0 (4.0–32.0)
Patient dialyzed, *n* (%)	–	0	0	0	0
Pulmonary abnormalities, *n* (%)	–	0	0	0	2 (40.0)
Anti-cytokine therapy					
Systemic anti-cytokine therapy given, *n* (%)	–	0	1 (14.3)	2 (50.0)	4 (80.0)
Tocilizumab	–	–	1 (14.3)	1 (25.0)	4 (80.0)
1 dose	–	–	1 (14.3)	1 (25.0)	3 (60.0)
2 doses	–	–	0	0	1 (20.0)
Corticosteroids	–	–	0	2 (50.0)	2 (40.0)
Other	–	–	0	0	0

Only the first CRS episode is summarized for each patient. Time to onset of CRS is since first tisagenlecleucel infusion. Time to ICU admission is since first tisagenlecleucel infusion

Patients: *n* = 42, Studies: NCT01747486, A2201 and NCT01029366, B2102J

*CLL* chronic lymphocytic leukemia, *CRS* cytokine release syndrome, *ICU* intensive care unit, *SD* standard deviation

*All percentages below are based on the number of patients with corresponding CRS grades

## Conclusions

CRS is a unique, expected, on-target toxicity directly related to the mechanism of action of CD19-targeted CAR T cell therapy. Although most patients receiving CD19 CAR T cell therapies had mild CRS, some developed significant symptoms requiring ICU-level care. In patients with ALL receiving CAR T cell therapy, CRS severity was related to disease burden [7], whereas this association was less obvious in initial experience with patients with CLL and NHL. Several groups have found the CTCAE system to be inadequate for grading CAR T cell therapy–associated CRS, and new grading scales have been proposed [41]. We developed the Penn grading scale, which distinguishes between levels of CRS-related care and between life-threatening and non-life-threating events.

It is important to highlight that the Penn grading scale is based primarily on clinical parameters and not laboratory values of inflammatory markers. Although laboratory blood tests are important, inflammatory biomarkers such as ferritin, CRP, and serum cytokines, including IL-6, are not included in the Penn grading scale. A large analysis of these markers showed strong association of peak value of parameters such as CRP and IL-6 with CRS, but they did not predict CRS when assessed early and did not add to clinical grading [43]. In addition, most US hospitals do not currently have access to rapid-turnaround cytokine measurements. Biomarker-based grading systems depend on the quality of laboratory tests, which may be affected by the types of testing kits available at each hospital. Grading scales based on specific clinical parameters can be applied more widely and used by many clinical trial sites and hospitals. This is important in the case of CRS caused by CAR T cell therapy, which can occur several days after infusion and therefore may be treated at a local hospital rather than the institution where the infusion was performed. An additional benefit of the Penn grading scale being based on clinical symptoms is that it allows for uniform data reporting across clinical trial sites, disease indications, and treatment modalities. We believe this grading scale is generalizable because it is based on easily identifiable clinical symptoms and can be effectively and broadly implemented at academic teaching institutions as well as local hospitals, providing guidance for physicians, nurses, and clinical pharmacists involved in managing patient care. We have now shown that this scale can be successfully used by multiple US and global sites participating in tisagenlecleucel trials [8, 9, 20, 44].

The extension of CAR T cell therapy beyond specialized academic medical centers requires an understanding of CRS pathogenesis and clinical manifestations, reproducible CRS grading, and comparison of CRS across trials and treatment platforms. To address this, the Penn grading scale can be, and has been, applied globally in multicenter trials and across different platforms; such a scale will allow researchers, physicians, and health authorities to better describe and compare CRS toxicities, levels of care, and interventions in patients receiving T cell-directed therapies. Our experience confirms that such a system can be effectively used in multicenter trials and implemented across several indications for numerous diseases. Consistency in grading will provide a major advance in our ability to compare toxicities of different cellular therapies in the absence of randomized trials.

## Abbreviations

ALL: Acute lymphoblastic leukemia
CAR: Chimeric antigen receptor
CHOP: Children’s Hospital of Philadelphia
CLL: Chronic lymphocytic leukemia
CR: Complete response
CRP: C-reactive protein
CRS: Cytokine release syndrome
CTCAE: Common Terminology Criteria for Adverse Events
DLBCL: Diffuse large B cell lymphoma
ICU: Intensive care unit
LFT: Liver function test
mAB: Monoclonal antibody
MM: Multiple myeloma
NCI: National Cancer Institute
NHL: Non-Hodgkin lymphoma
Penn: University of Pennsylvania
r/r: Relapsed or refractory
TNF: Tumor necrosis factor
