# Correction to: Grading of cytokine release syndrome associated with the CAR T cell therapy tisagenlecleucel

**DOI:** 10.1186/s13045-018-0627-z

**Published:** 2018-06-13

**Authors:** D. Porter, N. Frey, P. A. Wood, Y. Weng, S. A. Grupp

**Affiliations:** 1 Division of Hematology-Oncology, Blood and Marrow Transplantation and Cellular Therapy Program, Perelman School of Medicine and Abramson Cancer Center, University of Pennsylvania, Philadelphia, PA USA; 2 Division of Hematology-Oncology, Blood and Marrow Transplantation and Cellular Therapy Program, Perelman School of Medicine and Abramson Cancer Center, University of Pennsylvania, Philadelphia, PA USA; 3 Department of Pediatrics, Perelman School of Medicine, University of Pennsylvania, Philadelphia, PA USA; 4 Department of Pediatrics, Children’s Hospital of Philadelphia, University of Pennsylvania, Philadelphia, PA USA; 5 Division of Oncology, Center for Childhood Cancer Research and Cancer Immunotherapy Program, Children’s Hospital of Philadelphia, 3501 Civic Center Blvd. CTRB 3006, Philadelphia, PA 19104 USA

## Correction

The original article [1] contains several small errors. The errors & concurrent corrections are listed below:

1. Under the ‘CRS: a clinical overview’ sub-heading of the background, the sentence at the end of the second paragraph should instead read as the following:

‘The ability to discontinue the drug before prolonged CRS occurs, the use of CTCAE grading, and the potency of the therapy likely all contributed to the low rates of CRS observed (2% of patients experienced grade 3 CRS).’

2. Under the ‘CTCAE scale’ sub-heading of the background, the sentence at the beginning of the section should instead read as the following:

‘The CTCAE CRS grading scale was modified to define mild, moderate, severe, and life-threatening CRS, regardless of the inciting agent, and to guide treatment recommendations [36].’

3. In Table 2, the text that is descriptive of grade 4 on the 2014 lee et al. grading scale should instead read as the following:

‘Life-threatening symptoms. Requirements for ventilator support or grade 4 organ toxicity (excluding transaminitis)’.

4. Under the ‘A new CRS grading scale developed based on treating patients with tisagenlecleucel’ sub-heading of the background, the sentence beginning ‘Many cases of mild CRS…’ should instead read as the following:

‘Many cases of mild CRS resolve with minimal intervention.’

5. Under the ‘A new CRS grading scale developed based on treating patients with tisagenlecleucel’ sub-heading of the background, the sentence beginning ‘In patients with grade 2 CRS…’ should instead read as the following:

‘In patients with grade 2 CRS, there may be some signs of organ dysfunction, such as grade 2 creatinine or grade 3 liver function test (LFT) results, hospitalization for managing CRS symptoms (which may include management of fevers in the setting of neutropenia), or the need for intravenous therapies (such as antibiotics or other medications).’

6. Under the ‘A new CRS grading scale developed based on treating patients with tisagenlecleucel’ sub-heading of the Background, in the sentence beginning ‘For example, if a patient with r/r ALL develops hypotension…’, the word ‘recently’ at the end of the sentence should be disregarded.

7. Affiliation #3 should instead read as the following:

‘Department of Pediatrics, Perelman School of Medicine, University of Pennsylvania, Philadelphia, PA, USA.’

8. Reference [21] in the original article should instead read as displayed by reference [2] in this correction article.

9. Reference [26] in the original article should instead read as displayed by reference [3] in this correction article.

10. Reference [42] in the original article should instead read as displayed by reference [4] in this correction article.
